# Effects of Niacin Restriction on Sirtuin and PARP Responses to Photodamage in Human Skin

**DOI:** 10.1371/journal.pone.0042276

**Published:** 2012-07-31

**Authors:** Claudia A. Benavente, Stephanie A. Schnell, Elaine L. Jacobson

**Affiliations:** Arizona Cancer Center and Department of Pharmacology and Toxicology, College of Pharmacy, The University of Arizona, Tucson, Arizona, United States of America; University of Tennessee, United States of America

## Abstract

Sirtuins (SIRTs) and poly(ADP-ribose) polymerases (PARPs), NAD^+^-dependent enzymes, link cellular energy status with responses to environmental stresses. Skin is frequently exposed to the DNA damaging effects of UV irradiation, a known etiology in skin cancer. Thus, understanding the defense mechanisms in response to UV, including the role of SIRTs and PARPs, may be important in developing skin cancer prevention strategies. Here, we report expression of the seven SIRT family members in human skin. SIRTs gene expressions are progressively upregulated in A431 epidermoid carcinoma cells (SIRTs1 and 3), actinic keratoses (SIRTs 2, 3, 5, 6, and 7) and squamous cell carcinoma (SIRTs 1–7). Photodamage induces dynamic changes in SIRT expression with upregulation of both SIRT1 and SIRT4 mRNAs. Specific losses of SIRT proteins occur early after photodamage followed by accumulation later, especially for SIRT4. Niacin restriction, which decreases NAD^+^, the sirtuin substrate, results in an increase in acetylated proteins, upregulation of SIRTs 2 and 4, increased inherent DNA damage, alterations in SIRT responses to photodamage, abrogation of PARP activation following photodamage, and increased sensitivity to photodamage that is completely reversed by repleting niacin. These data support the hypothesis that SIRTs and PARPs play important roles in resistance to photodamage and identify specific SIRTs that respond to photodamage and may be targets for skin cancer prevention.

## Introduction

Niacin deficiency in humans causes pellagra, a disease characterized by severe sunlight sensitivity in exposed skin. In animal studies, niacin restriction decreases NAD^+^ and poly(ADP-ribose) metabolism altering p53 expression, increased skin sensitivity to UV radiation, increasing genomic instability, impairing cellular responses to DNA damage, and increasing cancer incidence [1], [2], [3], [4], [5], while niacin supplementation decreases photoimmune suppression and cancers [6]. In cultured human cells, niacin deficiency alters the expression of p53, induces inherent genomic instability, and reduces survival both in untreated cells and following exposure to solar simulated light (SSL) [7], [8], [9]. Conversely, niacin supplementation, leading to increased NAD^+^, allows cells to recover more efficiently after DNA damage [8]. In human subjects, preliminary data suggests that niacin may have protective effects against DNA damaging agents [10], while NAD content has been inversely correlated with malignant phenotype [8], [11]. Thus, niacin may be involved in limiting skin damage and consequently human skin cancer by affecting multiple mechanisms including reduction of DNA damage and optimizing cellular responses to DNA damage.

Two classes of NAD^+^ consuming enzymes whose functions need to be much better understood in UV responses in skin are the sirtuins (SIRTs) and poly(ADP-ribose) polymerases (PARPs). PARP−1/2 are evolutionarily conserved nuclear enzymes activated by DNA strand breaks [12], including those that occur subsequent to photodamage. PARP−1/2 use NAD^+^ as a substrate to synthesize poly(ADP-ribose) (PAR) and polyADP-ribosylate various nuclear proteins, thereby exerting a role in the DNA damage surveillance network [13]. Extensive data can be found on the effects of niacin deficiency and its effect on PARP inhibition in the presence of DNA damage induced by chemical agents [7], [13], [14], [15], [16] and PARP activation has been demonstrated in response to UV irradiation [16], [17], [18]. Several studies have attempted to demonstrate that inhibition of PARP following UV damage enhances the cytotoxic effects of UV, analogous to what has been shown with alkylating agents. However, no report has shown that PARP inhibition is rate limiting for survival following UV irradiation. In fact, one study suggests that PARP does not participate in the repair of UV-induced pyrimidine dimers [19]. Thus, the role of PARP−1/2 during recovery from UV damage is controversial and requires further study.

SIRTs are a family of NAD^+^-dependent protein deacetylases that remove acetyl groups from modified lysine residues, form 2-O-acetyl-ADP-ribose as a product, and regulate the biological function of numerous target proteins, many of which are not yet identified [20]. In humans, there are seven known SIRT family members (SIRT1–7) with diverse cellular locations effecting roles in a wide range of cellular functions, including epigenetic gene silencing, DNA repair and recombination, cell cycle regulation, apoptosis, and regulation of metabolism [21], [22], [23], [24]. SIRT1, a nuclear enzyme and the most extensively studied family member, has been described as a guardian against cellular oxidative stress and DNA damage and, potentially, a tumor suppressor [24], [25]. Additionally, SIRT1 interacts directly with PARP−1, a nuclear protein activated by DNA breaks, to negatively regulate its activity via deacetylation [26], [27]. The spectrum of biological functions for SIRTs discovered to date is limited but warrants a thorough investigation, especially in skin tissue, where previous reports have focused on SIRT1 [28], [29], [30], [31] but implicate SIRTs in responses to UV, differentiation and tissue remodeling, which is important in maintaining skin homeostasis during the continual renewal of the epidermis.

Here, we have controlled cellular NAD^+^ by niacin restriction and demonstrate a hypersensitivity to photodamage that involves specific PARPs and SIRTs and accumulation of acetylated proteins, all of which are reversed by niacin repletion. These data show that niacin functions through NAD^+^ dependent photodamage response pathways that limit skin photodamage and thus, ultimately, skin cancer.

## Materials and Methods

Samples from 3 actinic keratosis (AK) and 5 squamous cell carcinoma (SCC), with respective normal adjacent skin biopsies were obtained from the project entitled “Chemoprevention of Skin Cancer Program Project Archived Samples for Additional Analyses”, reviewed by the University of Arizona Institutional Review Board (Tucson, AZ), Project Number 08-0201-04. The study from which the samples were obtained was conducted according to the Declaration of Helsinki Principles. Written informed consent was obtained from all subjects.

### Cell Culture

Normal human keratinocytes (NHEK, Lonza, Switzerland) were cultured in Keratinocyte Medium 2 containing KGM-2 SingleQuots (Lonza) and 0.07 mM calcium. Normal human diploid fibroblasts (CF3, Oklahoma Medical Research Foundation, Oklahoma City), human epithelial carcinoma cell line (A431, ATCC, Manassas, VA) and the established cell line of human epidermal keratinocytes (HaCaT cells, a gift from Dr. Norbert Fusenig, German Cancer Research Center, Heidelberg, Germany [32]), were cultured in Dulbecco’s Modified Eagle Medium (DMEM) containing 10% fetal bovine serum and kept in a humidified atmosphere containing 5% CO2 at 37°C. For niacin restriction, cells were grown as previously described [7].

### Extraction and Assay of NAD(H) and NADP(H)

NAD^+^, NADP^+^, NADH and NADPH were extracted from cultured cells by homogenization in 1.0 M NaOH. For total NAD and NADP determination, half of the samples were immediately neutralized with 2.0 M H3PO4. The reduced pyridine nucleotides were then oxidized by adding a 1/10 volume of 2.0 mM phenazine ethosulfate (Sigma Biochemical Co.). The sample was then brought to 1.0 M HClO4, incubated 10 min on ice, and the extract was centrifuged at 850× *g* for 10 min. The pellet was reserved for DNA quantification. The supernatant was neutralized with 1.0 M KOH, and the insoluble KclO4 was removed by centrifugation. The resulting supernatant was assayed for NAD^+^ and NADP^+^ as described previously [33], [34]. NADH and NADPH were extracted using the other half of each cell extract, which was heated to 60°C for 10 min to destroy oxidized pyridine nucleotides. The extract was neutralized with 2.0 M H3PO4, chilled and processed as described above for total NAD and NADP extraction and assay. NAD^+^ and NADP^+^ were calculated as the difference between total and reduced pyridine nucleotides. The pellet precipitated by HClO4 was dissolved in 0.5 M NaOH, and the DNA concentration was determined using the Quant-iT OliGreen Assay (Invitrogen). NAD(P)(H) values were normalized to DNA in each sample extracted.

### Epidermal Skin Reconstructs

Human keratinocytes were isolated from neonatal foreskin and grown in serum-free keratinocyte growth medium (KGM, Lonza) with 0.07 mM calcium. Keratinocytes from passage 2 were suspended in EPI-200-1 s medium (MatTek Corporation) and seeded in millicell insert (0.4 µM pore size, area of 0.6 cm^2^, Millipore) standing in wells of 24 well plates. 0.5·10^6^ cells in 0.5 mL volume were seeded inside of each insert and 1 mL of EPI-200-1 s medium was added outside of the insert. After two days of submerged culture, the medium inside of the insert was carefully aspirated and the cells were cultured at air-liquid interface by adjusting the medium level just up to the bottom of the inserts. The culture at air-liquid interface was maintained for 6 days and the cells were re-fed with medium from MatTek Corporation following the manufacture’s instruction. At the end of the culture the skin equivalents were harvested, fixed in buffered formalin and embedded in paraffin for immunohistochemistry. Paraffin-embeded sections were deparaffinized in xylene and hydrated in a graded series of alcohol. Sections were immersed in citric acid in a water bath at 87°C for 20 min to enhance antigen retrieval, rinsed in TBS and blocked in 3% BSA-TBS for 20 min. Immediately after blocking, sections were incubated in anti-filaggrin antibody (3137-500, Abcam) overnight at 4°C. Sections were washed in TBS, blocked once again, and incubated in 1∶25 Biotin-SP-conjugated AffiniPure Goat Anti-Rabbit IgG (Jackson ImmunoResearch Laboratories, West Grove, PA) for 30 min at 37°C.

### Irradiation

SSL treatment was performed as previously described by Wondrak et al. with a dose of 4.152 J cm-2 UVA and 193.8 mJ cm-2 UVB (25). Singlet oxygen (^1^O_2_) was generated as described previously [35] by photosensitization of 3.3 µM toluidine blue with exposure to visible light using a Sylvania 15-W Cool White light tube delivering light at an irradiance of 0.77 J/cm2.

### Cell Death Analysis

Cell death was determined using an apoptosis detection kit according to the manufacturer’s specifications (APO-AF; Sigma-Aldrich), as previously described [7], [36].

### Comet Assay and Quantification of DNA Damage

Alkaline single cell gel electrophoresis (comet assay), based on the method of Singh et al. [37], was performed as described previously [7]. Immediately prior to imaging, comet slides were hydrated and stained with ethidium bromide (1 mg/mL) for 15 min. Comets were analyzed using a fluorescence digital imaging system. Tail moments were calculated using Comet Assay Software Project (Casp) imaging software.

### Western Blot Analyses

Protein extraction for SIRT detection and acetylation profile was performed using RIPA buffer (50 mM Tris 50 mM, 150 mM NaCl, 0.1% SDS, 0.5% sodium deoxycholate, 1% NP40). Western blot analyses were performed using a 10 or 15% SDS polyacrylamide gel, followed by transfer to a PDVF membrane (Millipore). Antibodies used were anti-acetyl lysine (Ab193, Abcam, Cambridge, MA) at 1∶1000 dilution, anti-SIRT1 (NB100–2132, Novus Biologicals, Littleton, CO) at 1∶5000 dilution, 0.25 µg/mL anti-SIRT2 (ARP32384, Aviva Systems Biology, San Diego, CA), 0.25 µg/mL anti-SIRT3 (NB600–808, Novus Biologicals, Littleton, CO), 1 µg/mL anti-SIRT4 (NB100–1406, Novus Biologicals, Littleton, CO), 2 µg/mL anti-SIRT5 (ARP32390, Aviva Systems Biology, San Diego, CA), 2 µg/mL anti-SIRT6 (NB100–2524, Novus Biologicals, Littleton, CO), 0.5 µg/mL anti-SIRT7 (ARP32406, Aviva Systems Biology, San Diego, CA). The respective manufacturers provided antibody validation. In particular for SIRT4, SIRT4 validation was conducted using its overexpression in HEK293 cells and our Western blot analyses confirmed a single band at the predicted 38 kDa (Figure S1 and data not shown). Densitometric analyses were performed with Image J (NIH, http://rsbweb.nih.gov/ij/) using α-tubulin or β-actin expression to correct for protein loading.

### qPCR

Total RNA from cultured cells was prepared using the RNeasy Mini Kit purification system (Qiagen, Valencia, CA) according to the manufacturer’s instructions. Total RNA from skin tissue samples was prepared using the RNeasy Fibrous Tissue Mini Kit (Qiagen, Valencia, CA) following the manufacturer’s instructions. RNA integrity was assessed by inspection of the 18 S and 28 S ribosomal RNA bands using gel electrophoresis. DNA synthesis was performed with the TaqMan Reverse Transcription kit (Applied Biosystems, Foster City, CA) according to manufacturer’s instructions using random hexamers and 1 µg of total RNA. For TaqMan-based qPCR expression profiling, 25 ng of each cDNA was added to the iTaq Supermix (BioRad, Hercules, CA) along with the TaqMan MGB probes according to the manufacturer’s instructions (Applied Biosystems, Foster City, CA). qPCR was performed essentially as described [38]. TaqMan Gene Expression Assays purchased from Applied Biosystems were used to detect human SIRT1 (Hs0109006_m1), SIRT2 (Hs00247263_m1), SIRT3 (Hs00202030_m1), SIRT4 (Hs00202033_m1), SIRT5 (Hs00202043_m1), SIRT6 (Hs00213036_m1), and SIRT7 (Hs00213029_m1), where all primers span exon junctions. Real-time fluorescence monitoring was performed with the ABI Prism 7000 (Applied Biosystems, Foster City, CA). A no-template control was run for each of the primer sets on every plate. The manufacturer’s estimated amplicon sizes and presence of a single amplicon band were verified by agarose gel analysis (data not shown). Each sample was run in triplicate, with the number of independent experiments provided in the figure legends. SIRTs’ mRNA levels are expressed as the Ct differential (dCt) normalized to GAPDH in each cultured cell type. In these cell lines GAPDH expression is constant relative to 18 S rRNA and stable following photodamage (data not shown) and thus was deemed an appropriate control. dCt measurements compare the relative abundance of each message, where the higher dCt reflects a lower total mRNA content and, therefore, a less abundant message. All expression measurements were performed in triplicate using three independently generated cDNA samples. Alterations in gene expression were considered significant at 50% change (>1.5-fold or <0.67-fold) and p<0.05. For tissue samples, 18 S rRNA was used as the reference housekeeping since GAPDH is not a stable message in skin tissues.

### Immunocytochemical Detection of Poly(ADP-ribose) Polymer

Cells were washed in phosphate buffer saline (PBS) and fixed in 5% formaldehyde in PBS for 30 min at room temperature. Cells were then permeabilized with 0.4% Triton X-100 in PBS for 3 min at RT, washed with PBS and blocked with 3% BSA-PBS for 30 min at RT. Cells were then incubated for 1 hr at 37°C in the presence of an anti-PAR monoclonal antibody (SA-216, Biomol) at 1∶50 dilution, followed by 3 washes in PBS and incubation with a secondary antibody conjugated with fluorescein for 1 hr at 37°C. Nuclear DNA was counterstained with DAPI and cells were examined at 60X magnification in a fluorescence digital imaging system.

## Results

### All SIRT Genes are Transcribed in Human Skin Derived Cells

SIRT expression has been reported in various tissue types both in mice and humans, but not all seven SIRTs have been characterized in skin. In order to understand the potential role of SIRTs in the response of skin cells to photodamage, we first examined which members of the SIRT family are expressed in various human skin derived cells in culture. We performed qPCR analyses and measured SIRT mRNA expression with probes specific to the seven different SIRT family members (SIRT1–7). In Table 1 we show that all seven SIRT genes are transcribed in NHEK cells. We also assessed the gene expression profiles in two other human epidermal cell lines, HaCaT and A431, and show that all SIRT genes also are transcribed in these cells (Table 1). While most SIRTs mRNAs are present at levels similar to those observed in NHEK, HaCaT cells show significantly lower levels of SIRT2, while A431 cells show higher levels of SIRT1 and SIRT3 transcripts (Table 1).

**Table 1:** SIRT gene expression in human skin-derived cultured cells**.**

	NHEK	HaCaT		A431		CF3	
	dCt	dCt	Fold differenceto NHEK	dCt	Fold differenceto NHEK	dCt	Fold differenceto NHEK
	(Mean ± SEM)	(Mean ± SEM)	(Mean ± SEM)	(Mean ± SEM)	(Mean ± SEM)	(Mean ± SE004D)	(Mean ± SEM)
**SIRT1**	10.2±0.3	9.5±0.2	1.4±0.2	8.7±0.3	2.4±0.4*	9.6±0.3	1.5±0.1
**SIRT2**	8.9±0.2	10.0±0.3	0.5±0.1*	9.2±0.4	0.9±0.2	8.9±0.2	1.5±0.1
**SIRT3**	10.4±0.3	9.8±0.4	1.9±0.7	8.4±0.4	3.9±1.1*	9.7±0.4	1.7±0.3
**SIRT4**	16.0±0.3	16.6±0.4	0.7±0.1	16.3±0.3	0.6±0.1	15.2±0.5	2.0±0.2*
**SIRT5**	10.7±0.4	10.6±0.3	1.0±0.2	10.4±0.3	1.0±0.2	11.5±0.4	0.5±0.1*
**SIRT6**	9.4±0.2	9.5±0.4	0.8±0.1	10.2±0.4	0.7±0.1	10.0±0.3	0.7±0.1
**SIRT7**	9.1±0.3	9.3±0.4	0.6±0.1	8.6±0.4	1.6±0.3	10.7±0.5	0.4±0.1*

qPCR analyses of SIRT genes in cultures cells.

dCt = Ct differential to GAPDH. Difference from NHEK at *p<0.05 and a fold change of >1.5 or <0.67. n = 3 independent experiments analyzed in triplicate.

Actinic skin damage is not restricted to the epidermis. Dermal fibroblasts and matrix proteins are subject to direct photodamage by UVA and oxidative stress secondary to UV exposure that can lead to solar elastosis. We therefore analyzed SIRT expression in CF3 cells (Table 1). Similar to what we observe in epidermal cells, CF3 cells express all seven SIRTs. Notable quantitative differences between keratinocytes and fibroblasts were higher transcript levels of SIRT4 and lower transcript levels of SIRT5 and SIRT7 in fibroblasts (Table 1).

### All SIRT Proteins are Expressed in Skin Cells

Next, we assessed whether SIRT transcripts in epidermoid cells are translated into protein using Western Blot analyses. All SIRT proteins are present in the epidermal (NHEK, HaCaT, and A431) (Figure S1) and dermal (CF3) human cells tested (data not shown). In HaCaT cells, we observe that the relative protein content of all SIRTs is approximately twice that in NHEK (Figure S1). However, the most notable difference in protein expression is in SIRT4, which is present at 3-fold and 4-fold higher levels in HaCaT and A431 cells, respectively, relative to NHEK. In CF3 cells, we observe that the relative protein expression levels are similar to NHEK, except for SIRT7, which is significantly reduced in these skin fibroblasts (data not shown).

### Both SIRT1 and SIRT4 Gene Expression Profiles are Upregulated by Photodamage

**Figure 1:**
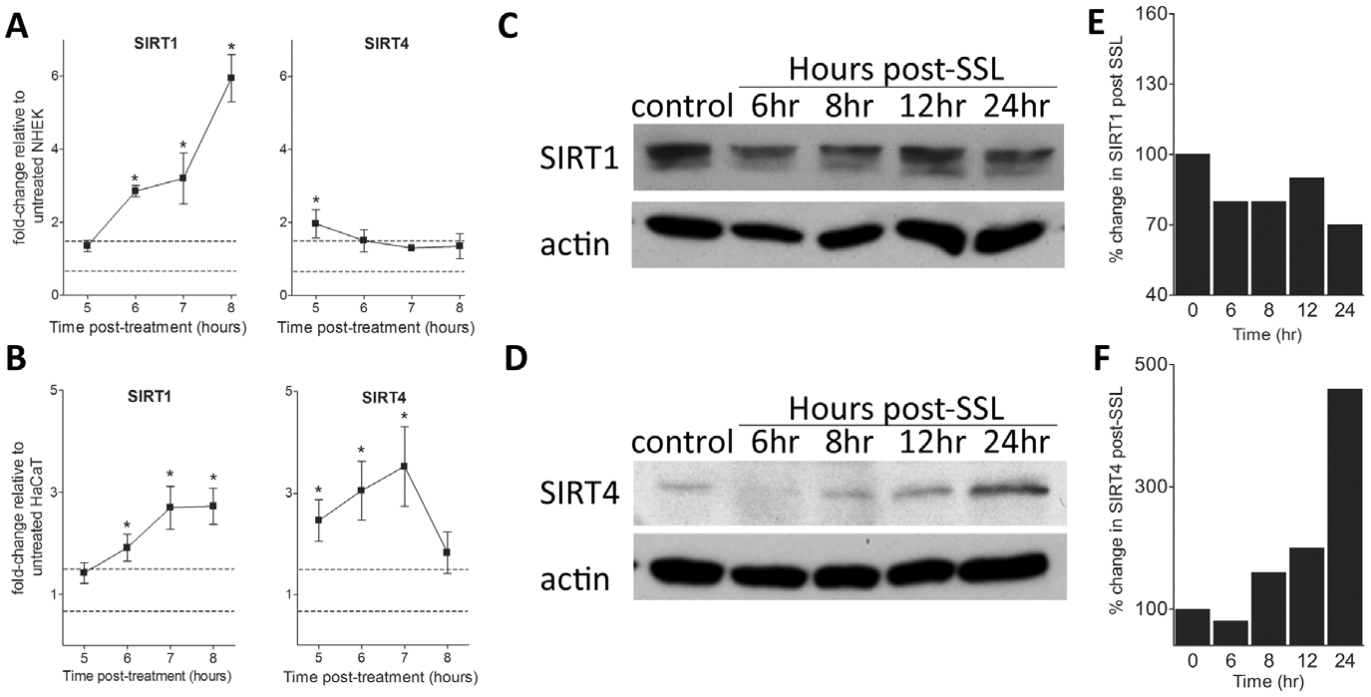
SIRT1 and SIRT4 gene and protein expression changes in NHEK and HaCaT cells following SSL treatment. (A–B) Time course of SIRT1 and SIRT4 gene expression after SSL treatment was measured using qPCR. (**A**) NHEK cells (mean ± SEM, n = 2 independent experiments, each ran in triplicate). (**B**) HaCaT cells (mean ± SEM, n = 3 independent experiments, each ran in triplicate). Gene expression levels shown are expressed as fold-change relative to untreated cells, where all samples were normalized to GAPDH gene expression. *p<0.05 and a fold change >1.5 or <0.67 (dashed lines). (**C**) SIRT1 and (**D**) SIRT4 protein expression in NHEK cells was detected by Western blotting using the antibody described in Materials and Methods. To normalize for protein loading, blots were probed with an antibody against α-tubulin. (**E**) Percentage change in SIRT1 and (**F**) SIRT4 protein expression level relative to untreated NHEK after normalization to α-tubulin.

Since photodamage is a primary initiator of pre-malignant and malignant lesions in skin, we examined the effects of SSL and singlet oxygen stress on SIRT transcripts in keratinocytes. We conducted preliminary experiments to determine the time frame of SIRT responses to SSL using qPCR (Figure S2). These data reveal that altered responses to treatment occur between 5 and 8 hours post SSL treatment for both gene transcripts. Figure 1 shows SIRT1 and SIRT4 mRNA expression in NHEK and HaCaT cells at 5 to 8 hours following SSL. We observe significant increases in SIRT1 expression beginning about 6 hours post treatment in both NHEK and HaCaT cells that increases up to 6-fold in NHEK and 3 fold in HaCaT cells by 8 hours. In addition, SIRT4 gene expression also is elevated in both cell lines 2-to 3-fold at 5 hours in NHEK and 5–7 hours in HaCaT cells post-treatment (Figure 1A and B). In contrast, transcription of all other SIRT genes is either mildly downregulated or unchanged following SSL (Figures S2 and S3).

We also analyzed protein expression profiles by Western Blot analyses in NHEK post-SSL treatment for all SIRTs. Figure 1C–F shows the results for the two SIRTs that had increased transcripts. We found that SIRT1 protein levels decreased approximately 30% at 6, 8, and 24 hours post treatment. However, SIRT4 protein content began to increase at 8 hours and by 24 hours was elevated nearly 500% compared to the untreated controls.

**Figure 2:**
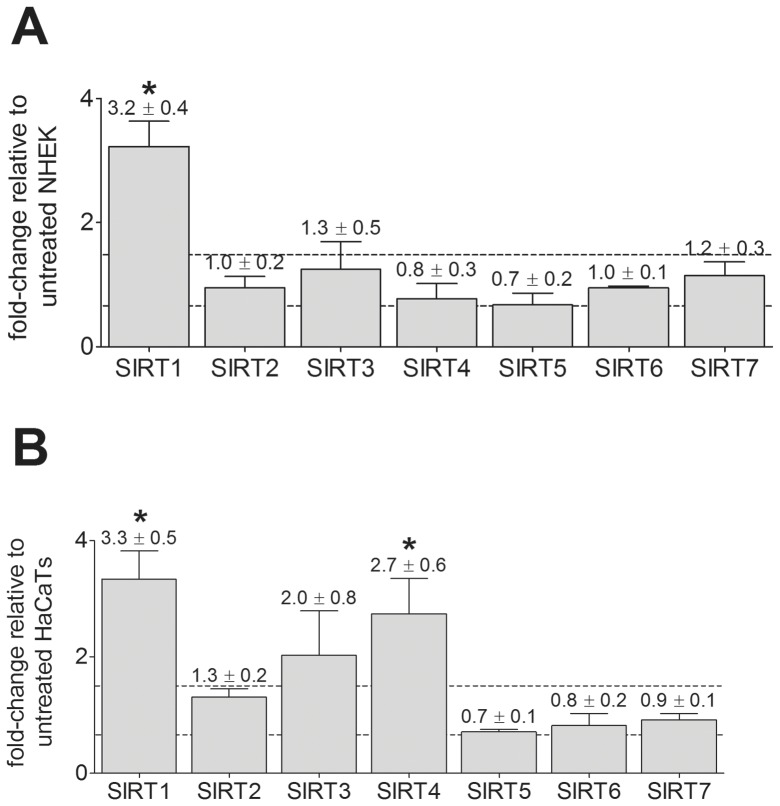
SIRT gene expression changes in keratinocytes following singlet oxygen stress. SIRT gene expression at 5 hours after singlet oxygen stress treatment was measured using qPCR. (**A**) NHEK (mean ± SEM, n = 2 independent experiments with triplicate samples). (**B**) HaCaT cells (mean ± SEM, n = 3 independent experiments with triplicate samples). Gene expression levels shown are expressed as fold-change relative to untreated cells, where all samples were normalized to GAPDH gene expression. *p<0.05 and a fold change >1.5 or <0.67 (dashed lines).

The effects of singlet oxygen stress, a well known form of indirect damage following UV exposure [39], which is generated by exposing the photosensitizer toluidine blue to visible light [40], were also examined for SIRT transcripts. Gene expression profiles following singlet oxygen stress were determined between 4 and 24 hours post-treatment as was performed for SSL. Results are shown only at 5 hours post-treatment, which is the time at which significant changes are observed. In NHEK, only SIRT1 transcripts increase in response to this dose of singlet oxygen stress, resulting in an increase in mRNA of 3.2 fold (Figure 2A). This increase in SIRT1 gene transcripts is reflected in an approximately 30% increase in SIRT1 protein levels at 6 hours post-treatment, which return to normal by 24 hours (data not shown). In HaCaT cells, singlet oxygen stress induced a significant increase in the transcripts of SIRT1 (3.3 fold) and SIRT4 (2.7 fold) mRNA (Figure 2B), similar to the effects observed with SSL.

### SIRT Transcript Levels are Altered during Induction of Keratinocyte Differentiation

**Figure 3:**
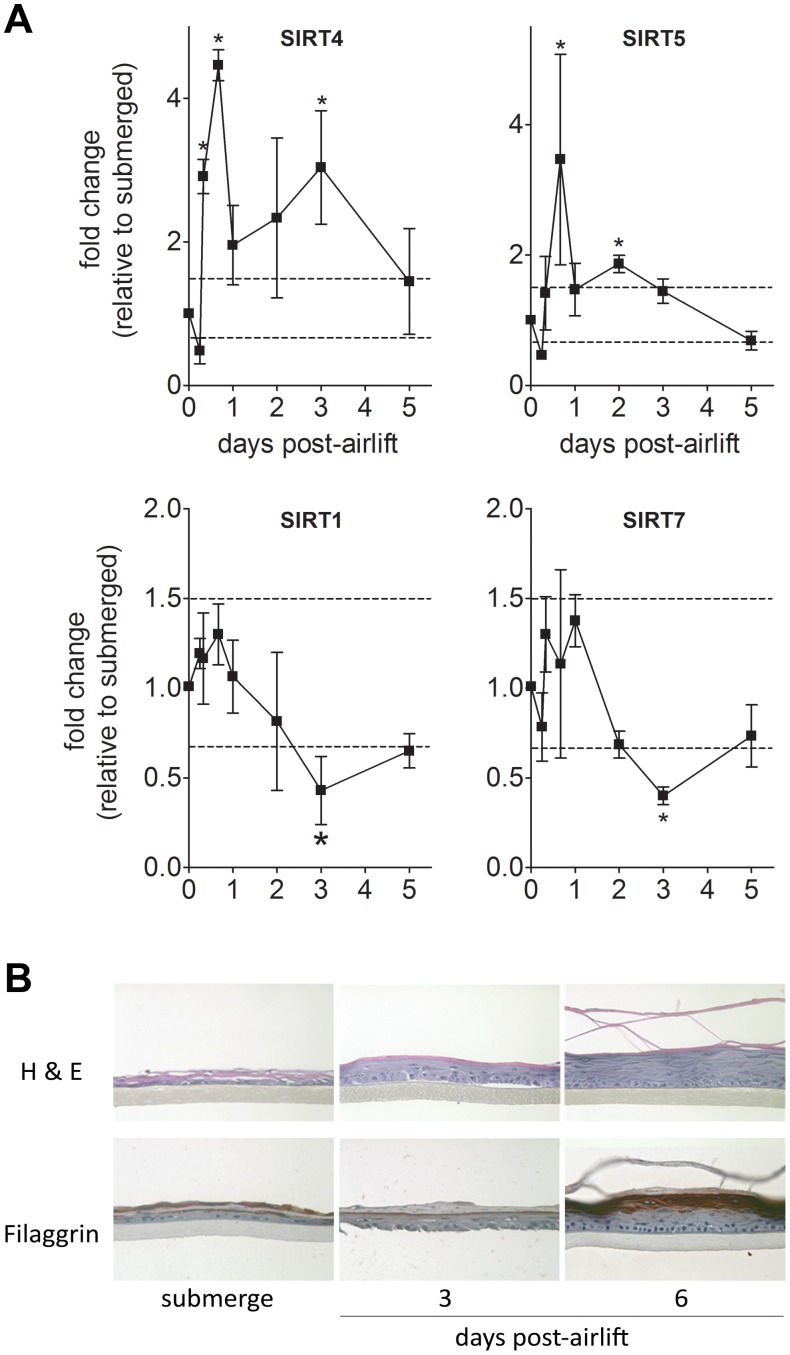
SIRT mRNA expression changes during keratinocyte differentiation. NHEK grown on a collagen-coated membrane were raised to the air-liquid interface (air-lift) to induce differentiation. (**A**) SIRT gene expression was measured by qPCR as a function of time. Expression levels shown are calculated as fold change relative to expression levels in submerged cultures (before airlift) after normalization to 18 S rRNA expression. Mean ± SEM, n = 4. *p<0.05 and a fold change >1.5 or <0.67 (dashed lines). (**B**) Hematoxylin and eosin (H&E) and filaggrin immunohistochemical staining of epidermal reconstructs reveals differentiation progress after airlift.

Since it has been shown that niacin, which elevates cellular NAD, the substrate for SIRTs, enhances terminal differentiation of the epidermis in humans [10], we next examined whether SIRT gene expression profiles are responsive to induction of epidermal differentiation in a skin reconstruct model in vitro. In this model, submerged, undifferentiated keratinocytes growing on a collagen matrix are raised to an air/liquid interface (airlift) resulting in the formation of a terminally differentiated epidermis in about 6 days [41]. We measured gene expression levels of SIRT1–7 at various times after induction of differentiation (Figure 3B). Figure 3A shows the dynamic responses of SIRT genes that changed compared to that of pre-induction conditions. We observe an early, transient upregulation of SIRT4 and SIRT5 transcripts after induction and a small decrease in gene expression of SIRT1 and SIRT7 at 3 days. No significant changes in mRNA expression are observed for the other SIRTs. However, as evidenced by the filaggrin staining (Figure 3B), the skin reconstructs represent a heterogeneous population comprised of undifferentiated and differentiated keratinocytes. Thus, the population of undifferentiated keratinocytes might mask the effect of differentiation on SIRTs expression. Nonetheless, multiple SIRTs are responsive to differentiation signals where an upregulation of SIRT4 and SIRT5 are early responses to induction and SIRT1 and SIRT7 are downregulated in the post proliferative stages of skin differentiation (Figure 3).

### All SIRT mRNAs are Upregulated in Human Squamous Cell Carcinoma

Based on the findings that SIRT1 and SIRT4 transcripts show dynamic responses to induction of differentiation, SSL, and oxidative stress, we examined the expression of SIRTs in human normal, premalignant (AK), and tumor (SCC) skin biopsies. SIRT transcript profiles from AK and SCC lesions were compared to paired adjacent normal tissue from the same subject. Table 2 shows that in SCCs, the gene expression levels of all seven SIRTs are 3-to 16-fold higher than in normal adjacent skin. In AKs, SIRT 2,3,5, 6 and 7 mRNAs are significantly upregulated while increases for SIRT 1 and 4 did not reach significance. These data add all 7 SIRTs to the list of genes whose expression is deregulated in SCC.

**Table 2:** SIRT gene expression in human biopsies.

	Human AK	SCC
	Fold difference relative to normal adjacent tissue	
	(Mean ± SEM)	
**SIRT1**	1.4±0.1	4.8±1.5*
**SIRT2**	3.5±1.6*	4.3±2.2*
**SIRT3**	5.7±2.8*	3.4±0.7*
**SIRT4**	2.9±2.3	4.5±1.0*
**SIRT5**	2.1±0.4*	8.4±2.6*
**SIRT6**	17.8±8.3*	8.7±2.8*
**SIRT7**	7.9±3.8*	15.7±7.4*

qPCR analyses of SIRT genes in skin biopsies.

Difference from normal adjacent tissue at *p<0.05 and a fold change of >1.5 or <0.67. n = 3 human biopsies each for AK and n = 5 for SCC, analyzed in triplicate.

### Niacin Restriction Leads to Hyperacetylated SIRT Target Proteins

Since we have shown low niacin status in human SCCs [8], we used our previously described model of niacin restriction [7], which modulates cellular NAD^+^ content, to block or limit SIRT activities to assess their function in response to photodamage. Consistent with previous studies, cells were depleted to approximately 10% of control NAD^+^ at 7 days of restriction and to about 1% by 14 days, yet cell division continues although at a slightly slower rate and the overall redox status of the cell is maintained (data not shown) [7]. We predicted that niacin restriction and consequent NAD^+^ depletion would lead to inhibition of the deacetylation activity of SIRTs, potentially providing a tool to identify novel SIRT targets. Using an antibody against acetylated lysine residues, we studied the protein acetylation profile of HaCaT cells from cultures grown in control and niacin restricted media and compared them to cells grown in the presence of reported SIRT inhibitors (sirtinol, splitomycin and 5 mM nicotinamide). Niacin-restricted HaCaT cell extracts accumulate significantly more acetylated proteins compared to control cells (Figure S4). Furthermore, niacin restriction is more effective than the SIRT inhibitors used in this experiment in causing accumulation of acetylated proteins. These results suggest that, as hypothesized, niacin-restricted cells have impaired SIRT activity, leading to hyperacetylated target proteins. This approach has the potential, when combined with proteomics and selective expression of SIRTs, to identify specific and novel SIRT targets following photodamage.

### Niacin Restriction Upregulates SIRT 2 and SIRT 4

**Figure 4:**
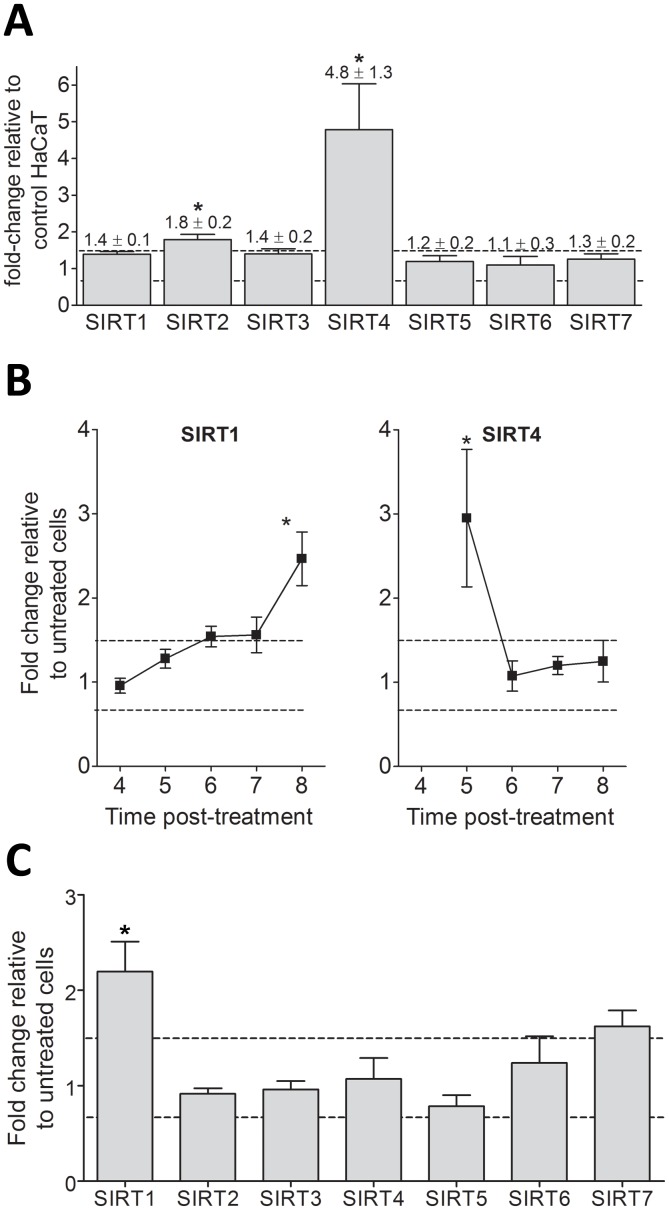
SIRT gene expression changes in HaCaT keratinocytes upon niacin restriction and photodamage. SIRT gene expression profiles in HaCaT keratinocytes were measured using qPCR. (**A**) Niacin-restricted SIRT gene message levels shown are expressed as fold-change relative to niacin replete HaCaTs. (**B**) Time course of SIRT1 and SIRT4 mRNA expression after SSL treatment. (**C**) Time course of SIRT gene expression after singlet oxygen stress. Expression levels shown for B and C are calculated as fold-change relative to untreated niacin-restricted HaCaTs. All samples were normalized to GAPDH gene expression. Mean ± SEM, n = 3 independent experiments with triplicate samples. *p<0.05 and a fold change >1.5 or <0.67 (dashed lines).

We next examined the effect of niacin restriction on SIRT gene expression and SIRT protein accumulation in niacin restricted cells using qPCR (Figure 4A) and Western Blot analyses (data not shown). SIRT4 and, to a lesser extent, SIRT2 gene expression is upregulated (Figure 4A), 4.8±1.3–fold and 1.8±0.2-fold, respectively. We could not detect changes in protein levels of any SIRTs due to niacin restriction.

### Niacin Restriction Sensitizes Cells to Photodamage

**Figure 5:**
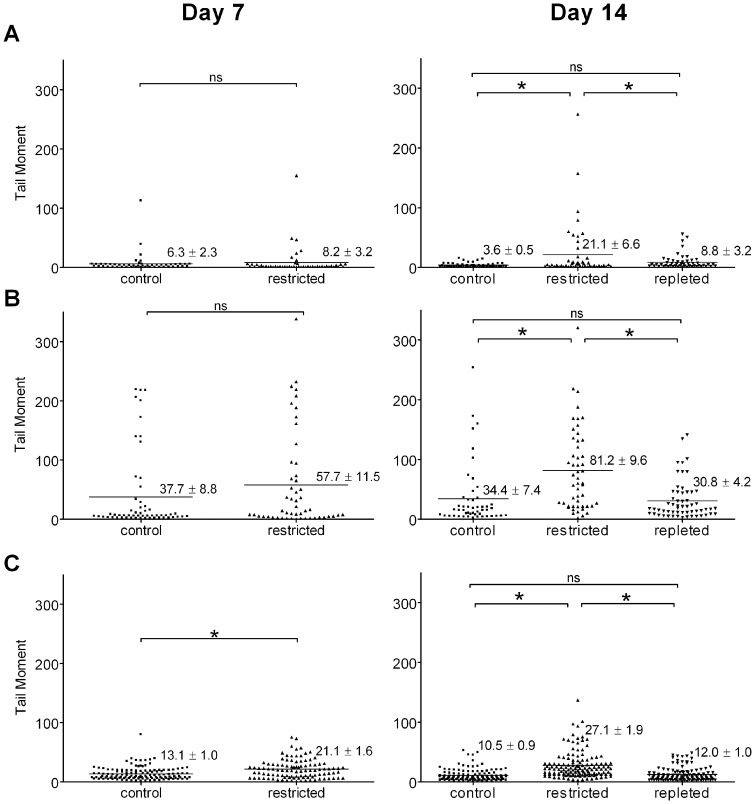
Effect of photodamage on DNA integrity in niacin-restricted HaCaT keratinocytes. DNA damage in HaCaT keratinocytes grown in the presence (control) or absence (restricted) of added niacin for 7 or 14 days was analyzed by alkaline comet assays. Dots represent the tail moment of single cells; mean tail moment is indicated by the lines shown for: (**A**) untreated, (**B**) SSL treated, and (**C**) singlet oxygen stress treated cells. *ns*, non significant difference; *, denotes significant differences, by unpaired Student’s t test, p<0.001.

We conducted experiments to determine the consequences of limiting NAD^+^-dependent responses to photodamage. We first performed alkaline comet assays on cells exposed to SSL or singlet oxygen stress, to determine effects of niacin restriction on DNA damage and repair. Niacin restricted HaCaT cells are significantly more sensitive to photodamage (Figure 5) where after 14 days, a significant increase in the sensitivity to treatment with SSL is observed, inducing an increase in mean tail moment from 34.4±7.4 in controls to 81.2±9.6 in restricted HaCaT cells (p<0.0001, Figure 5B). Following singlet oxygen treatment, at 7 days of niacin restriction, cells show a significant increase in mean tail moment from 13.1±0.9 to 21.1±1.6 (p<0.001, Figure 5C). Similar increases are observed at 14 days of restriction, where mean tail moments increase from 10.5±0.9 to 27.1±1.9 (p<0.0001, Figure 5C), but the increase in singlet oxygen induced tail moment is not significantly different when the magnitude of the increase in tail moment caused by niacin restriction alone at day 14 is taken into account.

To determine the consequences of the increased DNA strand breaks in niacin restricted cells, we measured cell survival following treatment with SSL or singlet oxygen stress. HaCaT cells after 14 days of niacin restriction show a significant increase in cell death both following SSL treatment (9.1±2.1% increase in controls versus 19.7±2.5% in restricted cells, p<0.05), and after singlet oxygen stress, which caused a 6.6±1.2% increase in cell death for controls versus 19.4±5.9% in restricted cells (p<0.05) (Table 3). As was observed with DNA strand breaks, the increased rates of cell death in restricted cells is entirely reversible (Table 3, Repleted) upon addition of niacin. Niacin repletion, which readily restores NAD^+^, reverses the increased sensitivity to SSL and singlet oxygen stress (Figure 5 and Table 3, repleted). The reversibility by niacin and resulting restoration of NAD^+^ suggests that the increased damage is due to reduced availability of NAD^+^, resulting in impaired function of NAD^+^-dependent responses to photodamage.

**Table 3:** Effects of photodamage on viability of niacin restricted HaCaT keratinocytes.

	Before treatment		24 hr post SSL		24 hr post ^1^O_2_	
	Day 7	Day 14	Day 7	Day 14	Day 7	Day 14
	% cell viability	% cell viability	% cell viability	% cell viability	% cell viability	% cell viability
**Niacin status**	(Mean ± SEM)	(Mean ± SEM)	(Mean ± SEM)	(Mean ± SEM)	(Mean ± SEM)	(Mean ± SEM)
**Control**	91.4±2.4%	93.6±1.9%	76.1±14.6%	83.4±6.4%	80.6±7.6%	85.9±5.7%
**Restricted**	91.1±3.9%	84.6±3.8%*	79.2±15.9%	66.4±9.5%*	79.1±15.9%	64.4±9.5%*
**Repleted**		93.7±0.5%		85.9±4.6%		88.7±5.0%

* p<0.05, n = 3.

Flow cytometry analyses of cell survival following photodamage.

**Figure 6:**
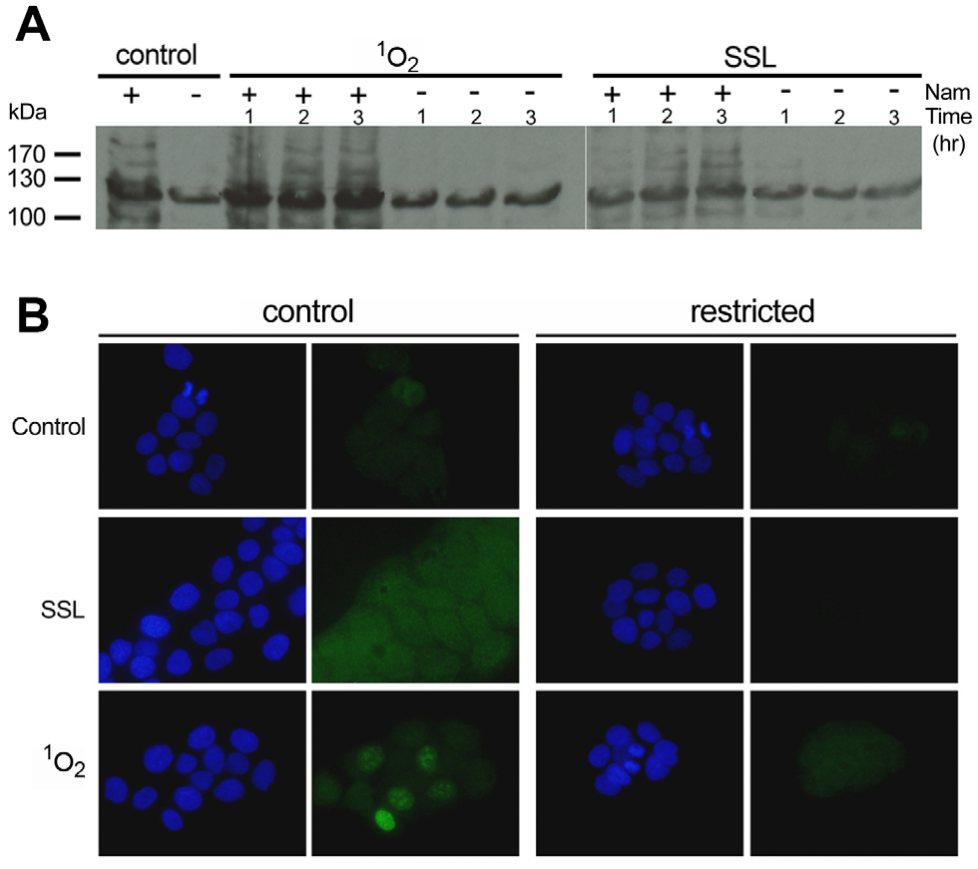
Effect of niacin restriction on PARP activation by photodamage. Polymer immunoblotting and immunocytochemistry of HaCaT keratinocytes grown for 14 days under control (+) or restricted (−) niacin (Nam) and exposed to photodamage. Cells treated with singlet oxygen stress (^1^O_2_) or solar simulated light (SSL) were analyzed by (**A**) Western blots using a PAR antibody and (**B**) immunostaining using a PAR antibody (green) and nuclear counterstaining with DAPI (blue).

### Poly-ADP-ribosylation is Impaired in Niacin-restricted Cells Following Photodamage

PARP activation and PAR accumulation are immediate responses following UV exposure; however, pharmaceutical inhibition of PARP following UV treatment has not been found to be rate limiting for survival as has been shown following treatment with alkylating agents. We tested whether niacin restriction in keratinocytes lowers NAD^+^ such that it is rate limiting for PARP activity. We have assessed PARP catalytic activation by detection of its product, PAR-modified proteins, in this model. PAR immunoblotting shows that SSL and singlet oxygen treatment result in an increase in PAR hypermodified proteins, including PARP−1, whose modifications appear as a ladder between 100–200 kDa [42], [43], [44] in keratinocytes grown in the presence of niacin (Figure 6A). The signal for PAR increases at 2 and 3 hours post SSL treatment and as early as 1-hour post singlet oxygen stress. In contrast, niacin restricted cells show reduced basal levels of PAR compared to control and no detectable PAR-hypermodified proteins are observed in response to SSL or singlet oxygen stress (modifications appearing above 130 kDa). This finding is further corroborated by PAR immunostaining of niacin-restricted cells compared to control cells following the same photodamage stress (Figure 6B). These data show that niacin restriction in keratinocytes does lead to cellular NAD^+^ concentrations that are rate limiting for poly(ADP)-ribosylation reactions following photodamage.

### SIRT Expression Profile Responses to Photodamage are Selectively Altered by Niacin Restriction

To determine how niacin restriction affects SIRT mRNA gene expression in response to photodamage, we exposed niacin restricted HaCaT cells to SSL or singlet oxygen stress and calculated the relative expression of SIRTs to untreated cells grown under niacin restriction (Figure 4B and C). Preliminary experiments were conducted to establish that the time frame where SIRT gene expression changes occur following photodamage is 4–8 hours post treatment (data not shown). We observe a SSL induced upregulation of SIRT1 and SIRT4 as was seen in keratinocytes that were not niacin restricted. This increase occurs in addition to the nearly 5-fold upregulation of SIRT4 by niacin restriction alone (Figure 4B), thus demonstrating a strong sensitivity of SIRT 4 mRNA expression to both SSL and niacin restriction stresses. Minor changes in other SIRTs can be seen in Figure S5. In response to singlet oxygen stress, niacin restricted cells upregulate only SIRT1 (Figure 4C).

## Discussion

We have previously reported that niacin restricted keratinocytes, which are consequently NAD depleted, accumulate intrinsic DNA damage, increased reactive oxygen species (ROS) and apoptotic cell death associated with DNA damage [7]. NAD^+^-dependent enzymes play a major role at the intersection between regulatory pathways of energy metabolism and cellular response pathways such as DNA damage and induction of cell death [2], [3], [4], [5], [45]. Both SIRTs and PARPs are NAD^+^-consuming enzymes involved in modulating cell homeostasis and survival or death at this intersection and are likely targets of niacin affecting these responses. PARPs rapidly consume NAD^+^ in response to stresses that result in DNA strand breaks presenting a unique challenge in photodamaged keratinocytes. Skin keratinocytes are chronically exposed to UV and in aging skin this exposure is elevated due to compromised stratum cornea. Furthermore, micronutrient status is decreased in aging skin. Thus, the combination of increased requirements and decreased availability of niacin as a substrate for NAD^+^-dependent photodamage response enzymes may contribute to energy stress that generates additional ROS and elevated genomic damage [7]. In this study, we examine the consequences of niacin restriction in keratinocytes subjected to photodamage stress to identify NAD^+^-dependent pathways important in photodamage responses that may be resistance factors in skin cancer development.

Since very little is known about SIRT expression and function in skin, we conducted and report here the first comprehensive analysis of gene and protein expression of all seven SIRTs in skin cells and tissues. We show that all seven SIRT family members are transcribed and translated in human dermal and epidermal cells and in human skin. We also have examined which SIRT transcripts are responsive to photodamage in keratinocytes. It has been reported previously that SIRT1 gene expression is upregulated following genotoxic stress in a number of different cell types [46], but this represents the first report of upregulation of SIRT1 gene expression after genotoxic stress in keratinocytes. Studies in other cell types suggest that SIRT1 functions in DNA repair and maintaining genomic stability [47] and new evidence begins to shed light on a possible mechanism where SIRT1 facilitates nucleotide excision repair by deacetylation of XPA [48].

In contrast to upregulation of SIRT1 transcripts, SIRT1 protein content has been reported to be reduced in keratinocytes following genotoxic stress introduced by UVB and hydrogen peroxide [49]. Our observation of decreases in SIRT1 protein following SSL (Figure 1C and 1E) is consistent with and extends that reported for UVB and hydrogen peroxide treatments in human skin keratinocytes [49]. The dynamic effects on SIRT1 transcripts and protein content following genotoxic stress suggest that multiple regulatory mechanisms are involved for SIRT1. One possible explanation for the observed effects is that upregulation of SIRT1 transcripts may be a response to the loss of SIRT1 protein that occurs following SSL treatment. With regard to SIRT1, our findings are consistent with prior reports but expand our understanding of the response to photodamage by examining both gene and protein expression time courses simultaneously after photodamage. However, throughout this study we observed a lack of correlation between SIRT mRNA expression and changes at the protein level for some of the SIRTs. This may be indicative of distinct regulatory mechanisms for each SIRT, for which further studies are warranted.

Our results also provide the first evidence that SIRT4 is responsive to genotoxic stress in skin as both SIRT4 transcripts and protein are increased post SSL treatment. We also report that niacin restriction in keratinocytes, which results in decreased NAD^+^ content, leads to upregulation of SIRT4 transcripts that are further upregulated when niacin restricted cells are subjected to SSL treatment. SIRT4 is a mitochondrial protein whose function(s) are still poorly understood. It has been proposed as a modulator of glutamate dehydrogenase in the regulation of insulin secretion in pancreatic beta cells [25] and as a regulator of fatty acid oxidation and mitochondrial gene expression in liver and muscle cells [50] where its expression seems to be highly integrated with SIRT1 mRNA and protein expression. The mechanism by which SIRT4 regulates its target proteins is presently controversial, as it has been reported to have protein-mono-ADP-ribosyl transferase activity instead of protein-deacetylase activity. However, kinetic studies demonstrating very low rates of mono-ADP-ribosyl transferase activity along with the observations that SIRTs can promote the hydrolysis of NAD^+^ in the absence of deacetylation substrates [51] generating the highly reactive ADP-ribose product which is known to modify proteins by non-enzymatic reactions [52] calls for further investigation of the physiological relevance of the mono-ADP-ribosylation reactions proposed for SIRTs. Despite the lack of understanding regarding the mechanism of SIRT4 activity, it appears to be emerging as an important regulator of mitochondrial oxidative metabolism and may function during stress to coordinate energy utilization. It is very interesting that not only do the stresses induced by niacin deprivation and photodamage increase SIRT4 expression individually, but when occurring simultaneously, result in an additive effect on the levels of SIRT4 upregulation.

Maintenance of skin homeostasis is dependent on the continual induction of terminal differentiation that regenerates the protective stratified skin barrier. Niacin has been shown to stimulate this process in humans [10]. Thus, we investigated whether SIRT family members may be involved in this process in skin, as SIRT1 and SIRT2 family members were previously reported to regulate terminal differentiation in other tissues [29], [53], [54], [55]. We have determined using an in vitro model of epidermal differentiation that SIRT4 transcripts, in particular, and to a lesser extent SIRT 5, are upregulated rapidly following induction of keratinocyte differentiation and that SIRT1 and SIRT7 mRNAs are downregulated late in the stages of the differentiation pathway after keratinocyte proliferation is complete. These preliminary findings point to a possible involvement of SIRT4 in this aspect of skin homeostasis and support further investigation of this hypothesis, which is the subject of another study.

Since we identified responses of specific SIRTs in skin cells to genotoxic stress and epidermal differentiation, we investigated whether other SIRT gene expression profiles are altered in precancerous (AKs) and malignant (SCC) human skin lesions. Previous studies have reported that SIRT1 is overexpressed in several cancer cell lines and cancerous tissue samples [56], [57]. We find that all SIRT transcripts are upregulated in SCCs and that many are upregulated in AKs. These data provide further impetus to investigate SIRTs as potential targets for maintaining skin homeostasis and potentially as skin cancer prevention targets.

A major new finding in these studies is the upregulation of SIRT4 gene transcripts under a number of conditions related to skin homeostasis: environmental (UV and oxidative stress), malignant transformation, and induction of differentiation. SIRT4 is a mitochondrial protein known to suppress the activity of glutamate dehydrogenase (GDH), at least in some cells, which would potentially favor glycolytic metabolism, commonly the primary source of energy for cultured cells and in many malignant cells [25]. We have shown that under niacin restriction, which leads to NAD^+^ depletion, HaCaT cells overexpress SIRT4 in the absence of DNA damage induction. Under these conditions, HaCaT cells utilize glutamine as an alternative energy source, requiring GDH and NADPH oxidase activity for recycling of NADP^+^ and for generation of TCA cycle intermediates. As a consequence, ROS are produced by NADPH oxidase [7]. It is possible that SIRT4 overexpression is a protective mechanism to avoid excess generation of ROS that could lead to exacerbated DNA damage and/or cell death. While in the above model niacin restriction decreases intracellular NAD^+^, low NAD^+^ also occurs following DNA strand break formation, which is known to activate poly(ADP-ribose) polymerases (PARP−1/2). Thus, modulation of GDH may be necessary transiently following DNA damage, dictating the upregulation of SIRT4. Elucidating the roles SIRTs play both directly in response to photodamage, or indirectly in response to altered NAD^+^ availability, has implications for understanding the maintenance of genomic integrity in skin following photodamage, and may be important to the understanding of early stages of carcinogenesis in skin.

Here, we show that niacin restriction increases the sensitivity to SSL and singlet oxygen stress in keratinocytes, as seen by the increase in DNA strand breaks and the increase in cell death rates. Since one of the most rapid responses to genotoxic stress is alteration of NAD metabolism due to activation by DNA strand breaks of PARP1/2, which convert NAD^+^ to PAR polymers, we assessed how niacin restriction affects PARP activity. Niacin restricted keratinocytes show reduced basal levels of PAR polymers compared to control and no detectable PAR-hypermodified proteins are observed in response to SSL or singlet oxygen stress. This observation lends support for role of PARP in UV responses, which has been a matter of controversy. In addition, we observe an increase in the acetylation state of the cells under niacin restriction, indicating that the activity of SIRTs is also inhibited under these conditions. The accumulation of acetylated proteins, DNA strand breaks, sensitivity to photodamage, and increased expression of SIRT2 and 4 exhibited by niacin restricted keratinocytes are completely reversible by addition of niacin to the culture medium that returns NAD^+^ content to control levels. These data point to niacin conversion to NAD^+^ as a critical factor in resistance to photodamage and suggest that elucidating the roles PARPs and SIRTs play both during altered NAD^+^ availability and in response to photodamage are essential for understanding mechanisms that maintain skin homeostasis and prevent skin cancer.

## Supporting Information

Figure S1 **SIRT protein expression in cultured epidermal cells**. SIRT protein expression was detected by Western Blot analyses using the SIRT antibodies described in Materials and Methods. To normalize for protein loading, all blots were probed with an antibody against α-tubulin. Numbers below each blot show the protein expression level relative to NHEK after normalization to α-tubulin. Numbers on the right depict molecular weight estimated from known molecular weight protein markers on the SDS page gel.
(TIF)

Figure S2 **SIRT gene expression changes in HaCaT cells upon SSL treatment.** SIRT gene expression profiles after SSL treatment were measured using qPCR. Gene expression levels shown are expressed as fold-change relative to untreated cells, where all samples were normalized to GAPDH gene expression. Mean ± SEM, n = 3 independent experiments with triplicate samples. *p<0.05 and a fold change >1.5 or <0.67 (dashed lines).
(TIF)

Figure S3 **SIRT gene expression changes in NHEK cells upon SSL treatment.** SIRT gene expression profiles after SSL treatment were measured using qPCR. Gene expression levels shown are expressed as fold-change relative to untreated cells, where all samples were normalized to GAPDH gene expression. Mean ± SEM, n = 2 independent experiments with triplicate samples. *p<0.05 and a fold change >1.5 or <0.67 (dashed lines).
(TIF)

Figure S4 **Protein acetylation accumulates in niacin-restricted HaCaT keratinocytes.** Acetylation of cellular proteins was studied using Western Blot analysis utilizing an antibody against acetyl-lysine. HaCaT keratinocytes were grown in normal (control), deficient (restricted) niacin medium, and normal medium treated with SIRT inhibitors: 25 µM sirtinol, 50 µM splitomycin or 5 mM Nam. Bottom: α-tubulin used as protein loading control for each condition. The large band at 70 kDa was identified as albumin using mass spectrometry analysis.
(TIF)

Figure S5 **SIRT gene expression changes in niacin-restricted HaCaT keratinocytes induced by SSL treatment.** Time course of SIRT mRNA expression after SSL treatment was measured using qPCR. Expression levels shown are calculated as fold-change relative to untreated niacin-restricted HaCaTs, were all samples were normalized to GAPDH gene expression. Mean ± SEM, n = 3 independent experiments with triplicate samples. *p<0.05 and a fold change >1.5 or <0.67 (dashed lines).
(TIF)
