# Supplementary figures and images for: Effects of Niacin Restriction on Sirtuin and PARP Responses to Photodamage in Human Skin
Source: PLoS One. 2012 Jul 31;7(7):e42276. doi: 10.1371/journal.pone.0042276 (PMC3409181; doi:10.1371/journal.pone.0042276)

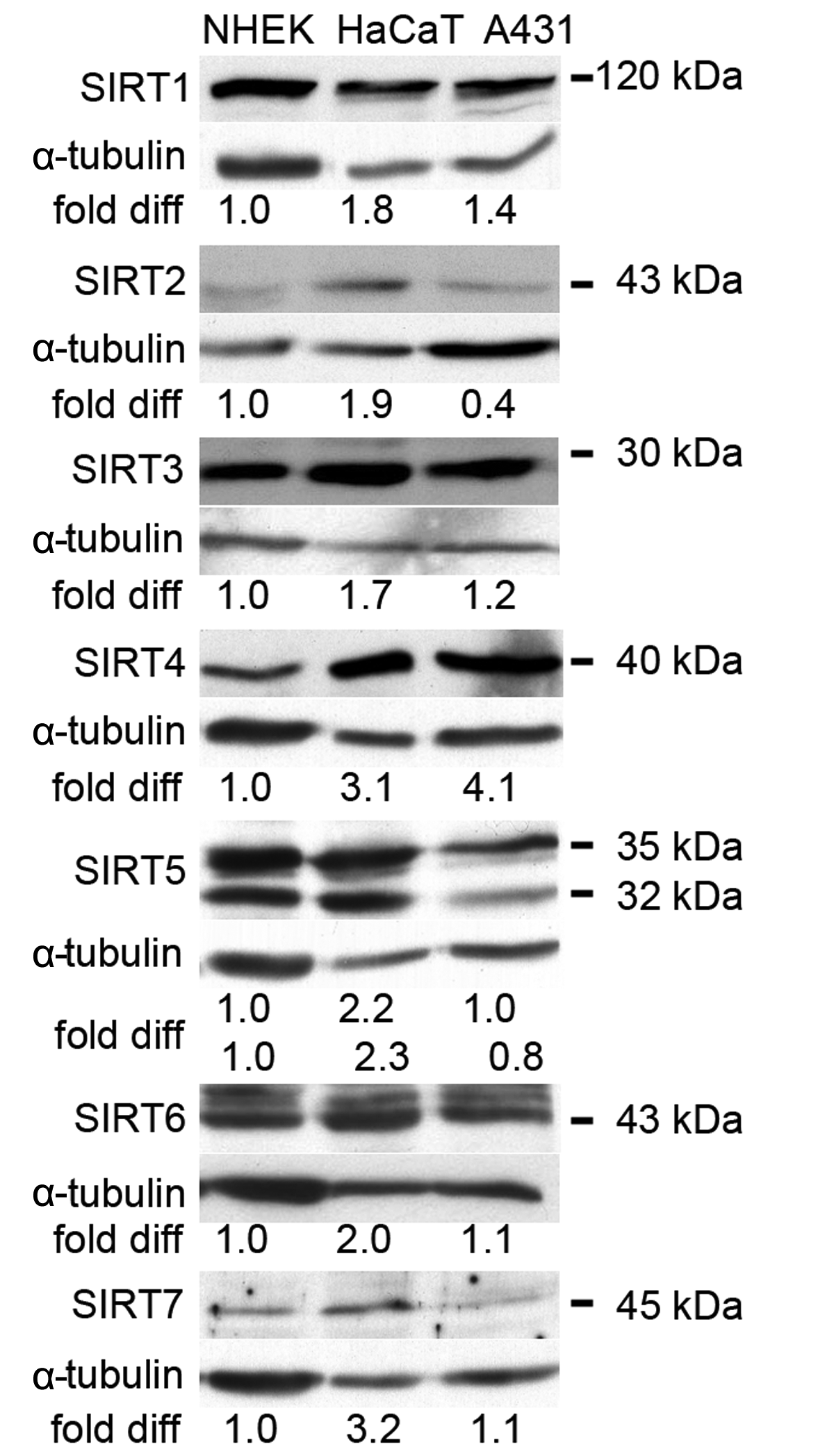

Supplement: Figure S1 — SIRT protein expression in cultured epidermal cells. SIRT protein expression was detected by Western Blot analyses using the SIRT antibodies described in Materials and Methods. To normalize for protein loading, all blots were probed with an antibody against α-tubulin. Numbers below each blot show the protein expression level relative to NHEK after normalization to α-tubulin. Numbers on the right depict molecular weight estimated from known molecular weight protein markers on the SDS page gel. (TIF) [file pone.0042276.s001.tif]

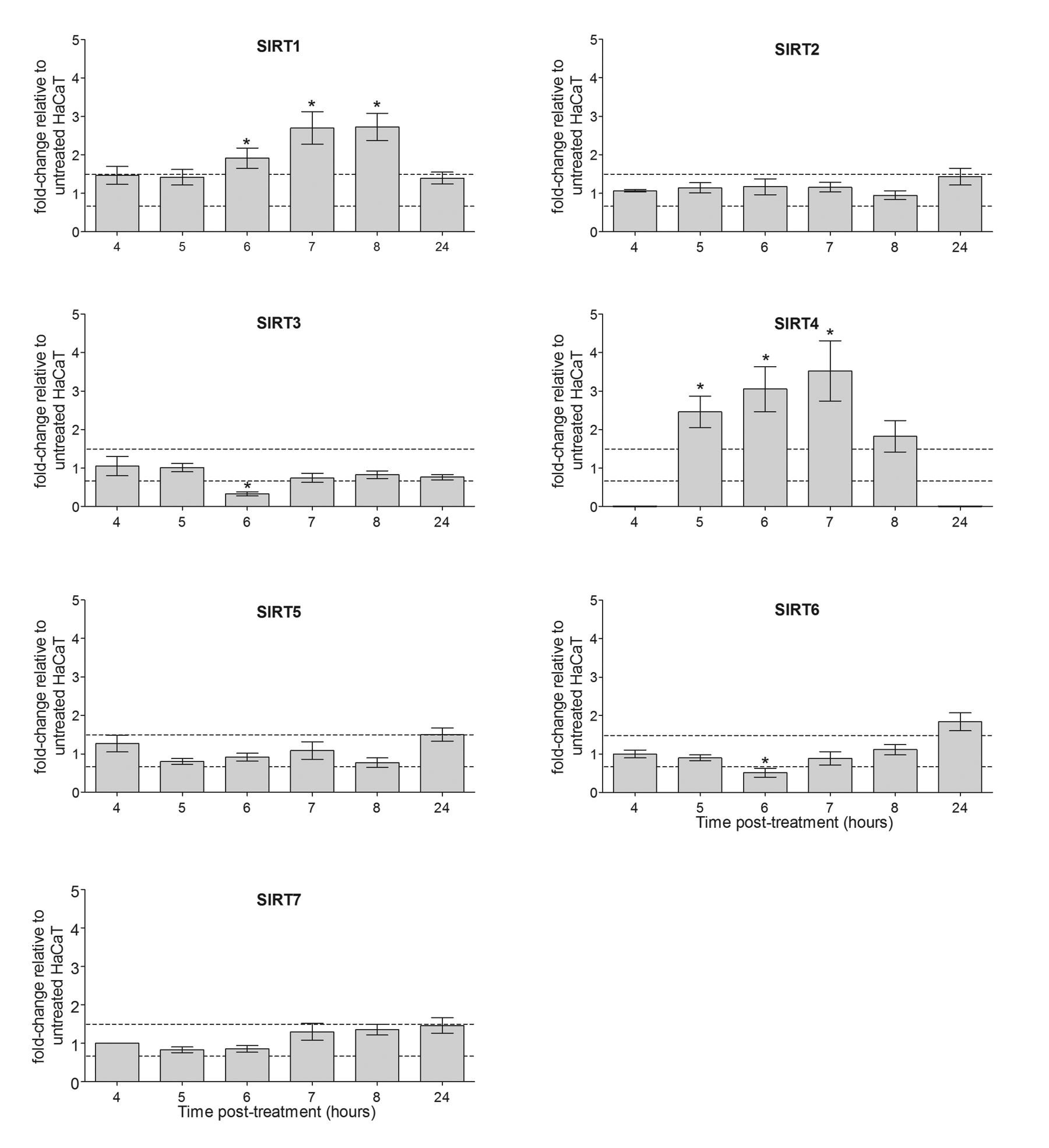

Supplement: Figure S2 — SIRT gene expression changes in HaCaT cells upon SSL treatment. SIRT gene expression profiles after SSL treatment were measured using qPCR. Gene expression levels shown are expressed as fold-change relative to untreated cells, where all samples were normalized to GAPDH gene expression. Mean ± SEM, n = 3 independent experiments with triplicate samples. *p<0.05 and a fold change >1.5 or <0.67 (dashed lines). (TIF) [file pone.0042276.s002.tif]

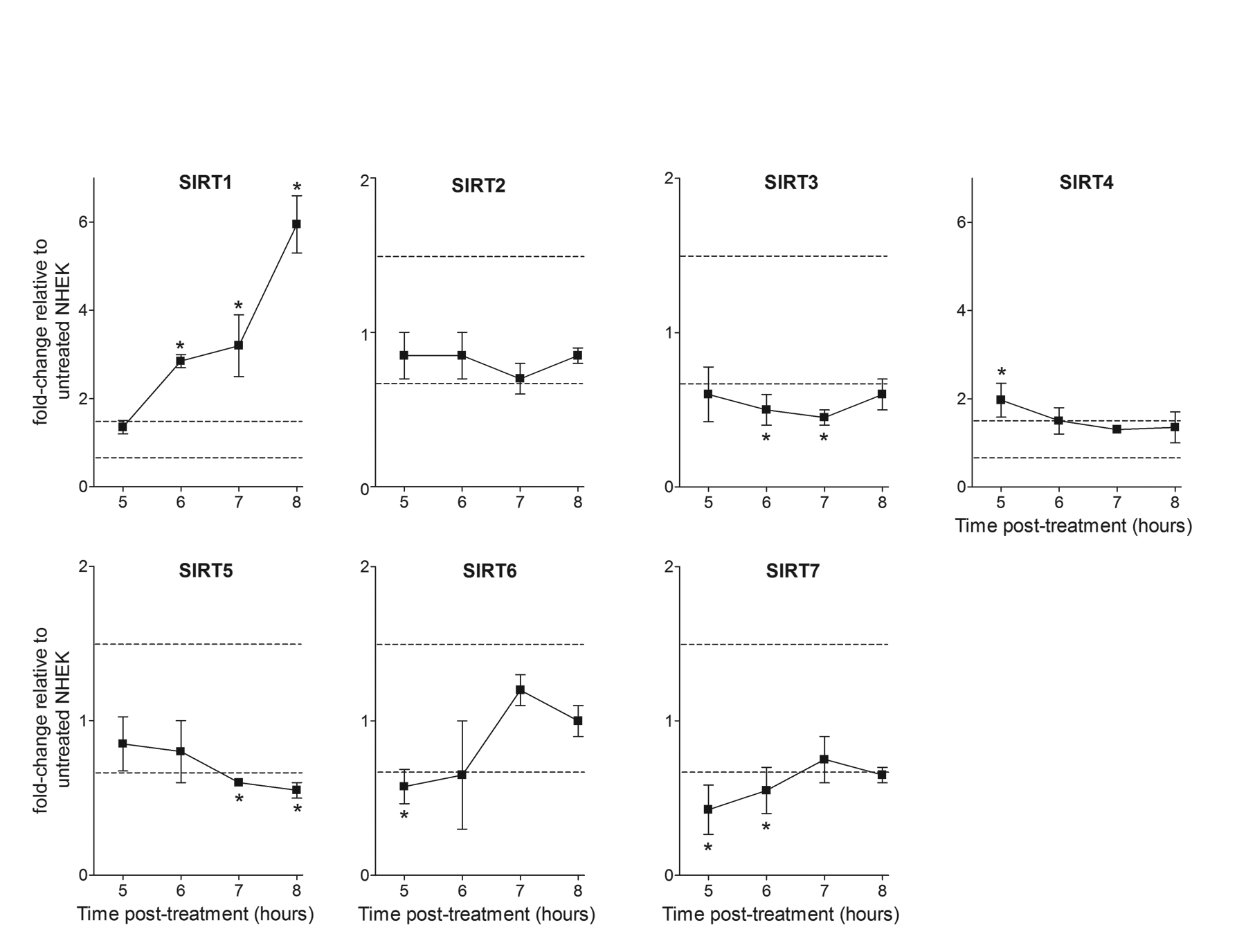

Supplement: Figure S3 — SIRT gene expression changes in NHEK cells upon SSL treatment. SIRT gene expression profiles after SSL treatment were measured using qPCR. Gene expression levels shown are expressed as fold-change relative to untreated cells, where all samples were normalized to GAPDH gene expression. Mean ± SEM, n = 2 independent experiments with triplicate samples. *p<0.05 and a fold change >1.5 or <0.67 (dashed lines). (TIF) [file pone.0042276.s003.tif]

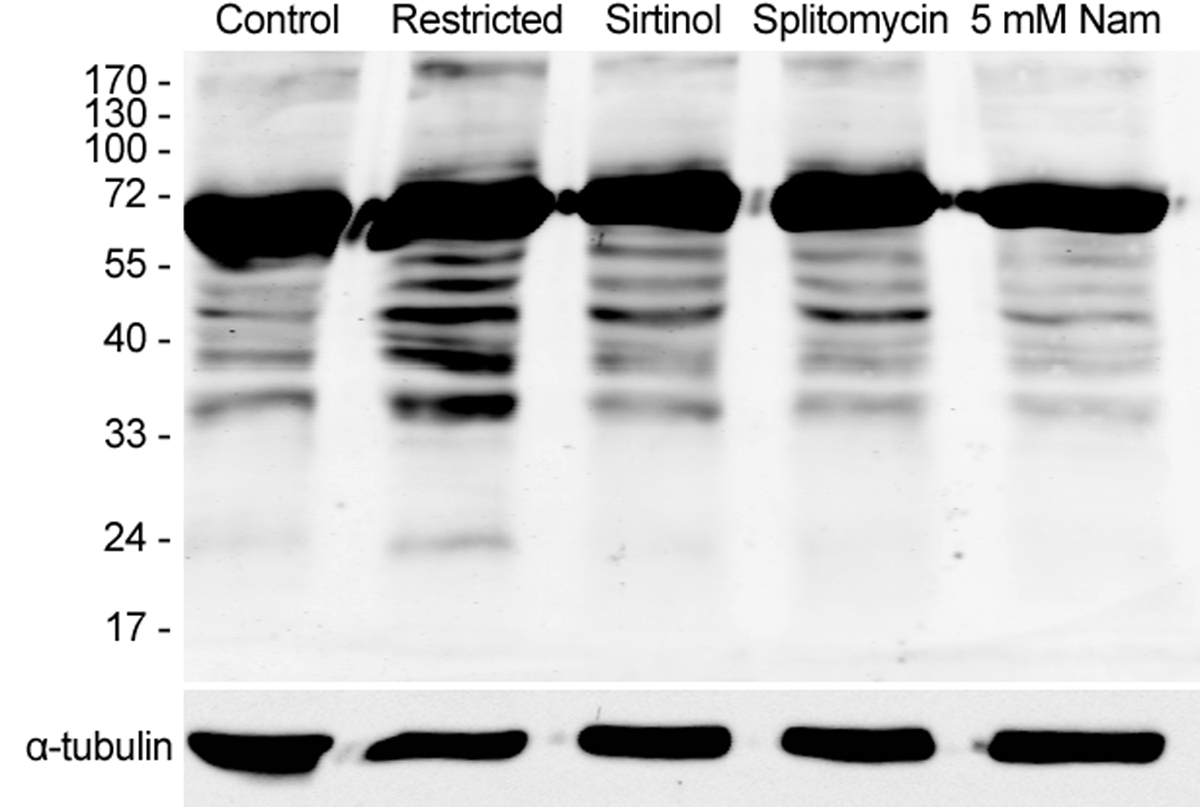

Supplement: Figure S4 — Protein acetylation accumulates in niacin-restricted HaCaT keratinocytes. Acetylation of cellular proteins was studied using Western Blot analysis utilizing an antibody against acetyl-lysine. HaCaT keratinocytes were grown in normal (control), deficient (restricted) niacin medium, and normal medium treated with SIRT inhibitors: 25 µM sirtinol, 50 µM splitomycin or 5 mM Nam. Bottom: α-tubulin used as protein loading control for each condition. The large band at 70 kDa was identified as albumin using mass spectrometry analysis. (TIF) [file pone.0042276.s004.tif]

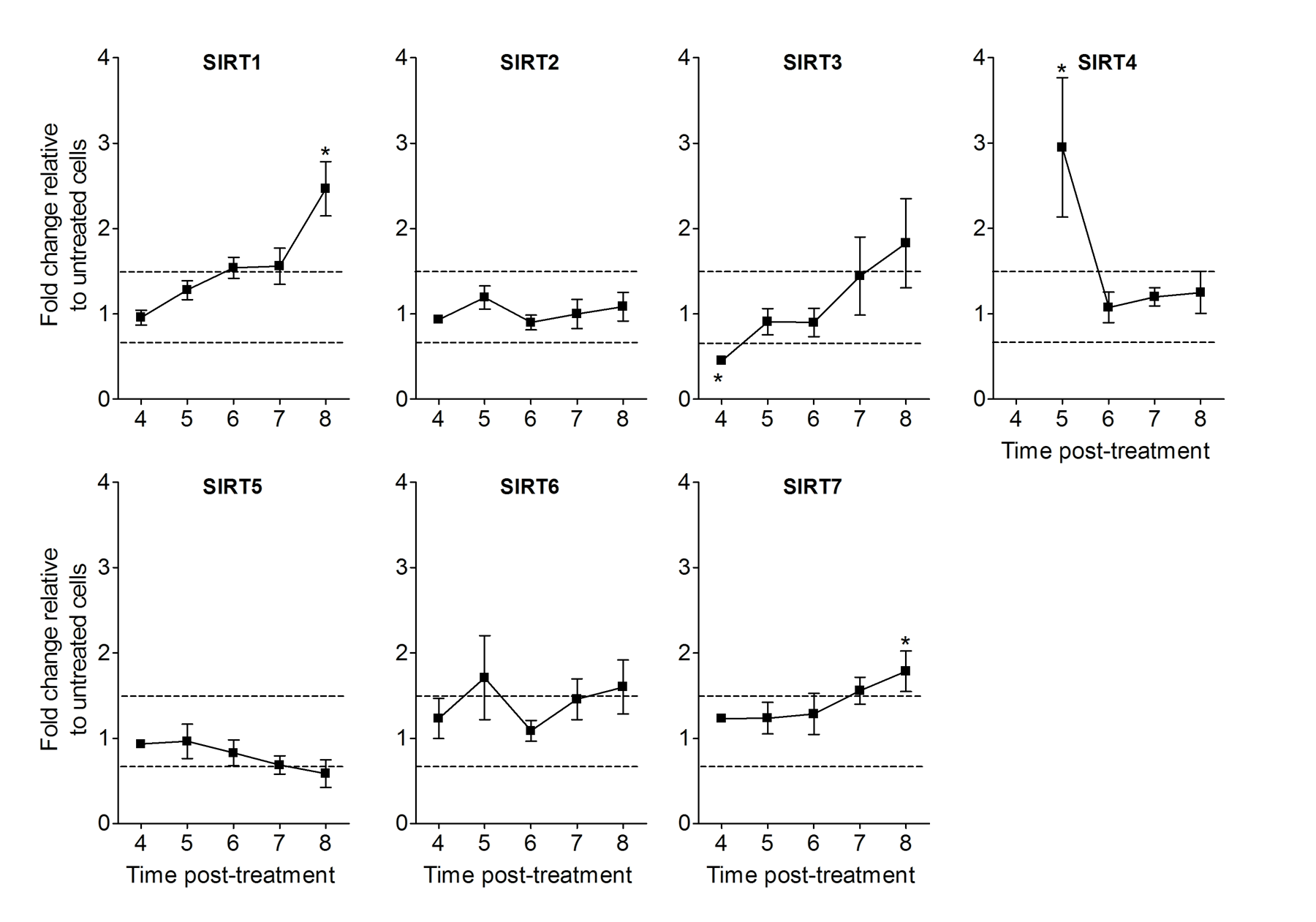

Supplement: Figure S5 — SIRT gene expression changes in niacin-restricted HaCaT keratinocytes induced by SSL treatment. Time course of SIRT mRNA expression after SSL treatment was measured using qPCR. Expression levels shown are calculated as fold-change relative to untreated niacin-restricted HaCaTs, were all samples were normalized to GAPDH gene expression. Mean ± SEM, n = 3 independent experiments with triplicate samples. *p<0.05 and a fold change >1.5 or <0.67 (dashed lines). (TIF) [file pone.0042276.s005.tif]
